# An easy spectrophotometric acid-base titration protocol for dissolved organic matter

**DOI:** 10.1016/j.mex.2022.101721

**Published:** 2022-05-05

**Authors:** Marawit Tesfa, Aline Dia, Malou Ollivier, Iván-David Osorio-Leon, Rémi Marsac

**Affiliations:** University of Rennes, CNRS, Géosciences Rennes - UMR 6118, Rennes, France

**Keywords:** Acid-base titration, UV-vis spectrophotometry, Dissolved organic matter

## Abstract

UV-vis spectrophotometric acid-base titration can characterize dissolved organic matter (DOM) acid-base properties. However, it requires incremental pH adjustment, which make the procedure time consuming and the results subjected to dilution effect. This study brings forth a new approach, referred as the “buffer method” for pH adjustments, by using carefully selected pH-buffers to adjust the pH. This, statistically validated method minimizes the pH adjustment time and lightens the laboratory work load. Chemical product cost associated with this novel method is slightly increased as compared to the previous approach, due to the necessity to use pH-buffers.

• Buffer method: Acid-base titration by using buffer for pH adjustment

• Buffer method validated by statistical means

• Rapid, reliable and economical method

## Graphical abstract

**Figure:**
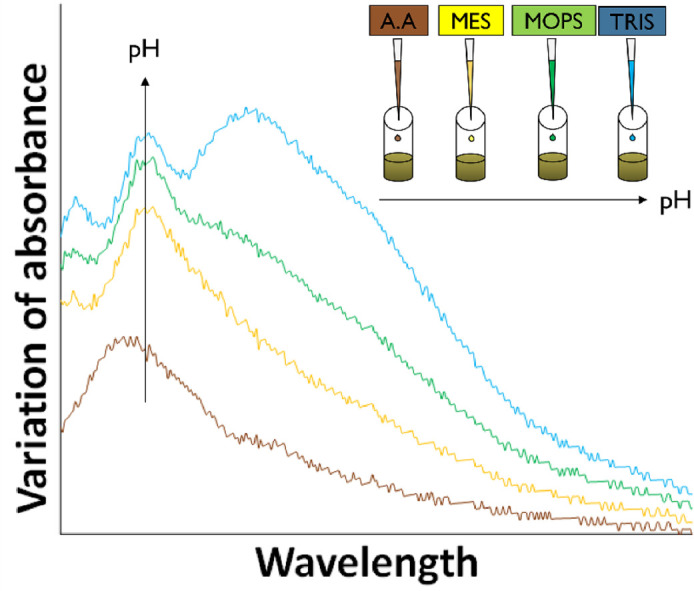

Specifications Table

**Table:** 

Subject area:	Environmental sciences
More specific subject area:	Wet chemistry
Method name:	“Buffer method”
Name and reference of original method:	Original method has no name in the literature, it was named in this article “Incremental method” References of original method [1]. In Situ Examination of the Protonation Behavior of Fulvic Acids Using Differential Absorbance Spectroscopy. Environ. Sci. Technol. 42, 6644–6649. https://doi.org/10.1021/es800741u [2]. Effects of Ionic Strength on the Chromophores of Dissolved Organic Matter. Environ. Sci. Technol. 49, 5905–5912. https://doi.org/10.1021/acs.est.5b00601 [3]. Using Spectrophotometric Titrations To Characterize Humic Acid Reactivity at Environmental Concentrations. Environ. Sci. Technol. 44, 6782–6788. https://doi.org/10.1021/es1012142 [4]. Comparison of the properties of standard soil and aquatic fulvic and humic acids based on the data of differential absorbance and fluorescence spectroscopy. Chemosphere 261, 128189. https://doi.org/10.1016/j.chemosphere.2020.128189
Resource availability:	All resources used in this study can be acquired from Sigma Aldrich and the International Humic Substances Society

Dissolved organic matter (DOM) is ubiquitous in aquatic systems and plays a key role in the environment as it is at the crossroad of multiple biogeochemical cycles. In particular, because of its capacity to form complexes with metal contaminants, the quantification of chemical entities that drive DOM-metal ion interaction is highly required [5], [6], [7], [8], [9], [10]. These are mainly acid-base functional groups, which can be determined by acid-base titrations. Amongst various approaches, UV-vis spectrophotometric acid-base titration allows the analysis of DOM acid-base properties at environmentally relevant concentrations [1], [2], [3], [4]. This can be used, for instance, to monitor changes of DOM deprotonation–protonation properties in water treatment processes [11].

The principle of spectrophotometric acid-base titration is to progressively increase the pH of the DOM solution with consecutive additions of small volumes of base. The absorbance of the solution is measured by spectrophotometry after each step of pH increment. Despite the simplicity of the method, it has not yet been automated, making the laboratory workload meticulous and time-consuming. In addition, as a consequence of all of these consecutive steps, this experimental procedure presents a significant dilution effect. This commonly used method will be referred to as the “incremental method”.

To lighten the laboratory workload and minimize the dilution effect with the "incremental method", we developed an alternative to the pH stabilization. Based on studies that have used buffers to keep the pH of DOM solutions constant without affecting the optical properties of the DOM [2,4], we proposed to generalize the buffer use to a wider range of pH, necessary for acid-base titration. The newly developed “buffer method” referred to as such further in this study, consists in dividing a DOM mother solution into aliquots, adjusting these aliquots pH individually with carefully selected buffers and measuring aliquots absorbance independently. Furthermore, this new protocol can be automated using an autosampler connected to the UV-vis spectrophotometer. The “buffer method” presents an automatable, faster experimental setup yielding results with minimum dilution effects for UV-vis spectroscopic acid-base titration of DOM.

**Reagents.** Suwannee River Fulvic Acid (SRFA, ref n° 3S101F) was acquired from the International Humic Substance Society (IHSS). This fulvic acid sample was selected because it is most commonly used and therefore, widely characterized. Potentiometric titration conducted by Ritchie and Perdue, [12] indicate that SRFA sample contains 12.00 mg L^−1^ of carboxylic groups and 1.48 mg L^−1^ of phenolic groups. The pH-buffer salts (Table 1), background salt (NaCl), acid (HCl) and base (NaOH) solutions were purchased from Sigma Aldrich. The pH-buffers are some of the widely used “Good's buffers” and acetic acid/acetate, which are available at relatively low cost (about 1€/g or below).

**Table 1:** Buffer solution properties used in this study

Buffer	Chemical formula	pKa	pH
TRIS	C_4_H_11_NO_3_	8,1	9.06
			8.62
			8.09
MOPS	C_7_H_14_NaO_4_S	7,15	7.44
			7.18
MES	C_6_H_13_NO_4_S	6,15	6.44
			6.11
			5.51
AA	C_2_H_4_O_2_	4,1	4.91
			4.48
			4.00

**Instruments.** Dissolved Organic Carbon (DOC) concentrations were determined by Shimadzu TOC-L carbon analyzer. The pH was monitored using HANNA, HI5221 pH meter. The pH meter was calibrated with standard HANNA buffers (4.01, 7, 10.1) and, new calibrations were performed for each buffer solution preparation. Absorbance was recorded, in a 1-cm-wide quartz cuvette, between 200 and 800 nm, with a UV-vis Shimadzu UV2600 spectrophotometer.

**Solution preparation.** A stock of DOM mother solution (1 g L^−1^) was prepared by dissolving 250 mg of organic matter powder into a total 250 mL volume of milliQ water and base (0.1 M NaOH). Complete dissolution was guaranteed by maintaining solution pH at 9 for 24 hours, with adjustments 3, 6 and 12 hours after beginning the dissolution procedure, using NaOH. The mother solution was diluted to 25 mg L^−1^ of carbon (mgC L^−1^) and the ionic strength (IS) to 0.01 M with background electrolytes (NaCl, 1 mol L^−1^). Buffer mother solutions were made by dissolving buffer salts in 40 mL ultrapure water. The pH of the buffer solutions was adjusted to the pKa value of the buffer or within a range of pKa ± 1 by using acid (HCl, 1 mol L^−1^) or base (NaOH, 1 mol L^−1^), then verified and readjusted if necessary after 3, 6, 12 and 24 hours. The final volume was raised to 50mL with ultrapure water, leading to a final buffer concentration of 1 mol L^−1^.

**Incremental titration protocol.** The “incremental method” [3] is illustrated in Fig. 1a and was carried out as follows: a 50 mL volume of the DOM mother solution was placed in a 100 mL plastic polypropylene beaker with a magnetic stirrer and under N_2_ bubbling throughout the titration process. The pH of the DOM solution was adjusted to 3 using HCl (0.1 mol L^−1^). The quartz cuvette was filled with 3 mL of the solution and the UV-vis spectrum was recorded. The 3 mL were dropped back to the beaker to avoid any variation of the volume of the titrated DOM solution. The pH of the titrated solution was subsequently raised to 3.2 by manual addition of a small volume of NaOH (0.1 mol L^−1^). After pH stabilization and recording, the quartz cuvette was first rinsed with the DOM solution then the absorbance of DOM was measured by spectrophotometry, and then put back into the beaker. Spectra were measured every ca. 0.2 pH units between 3 and 9. The volume of each base addition was recorded to later be considered for dilution calculations. Considering the pH stabilization and the pH modification steps (0.2 unit), this experimental process takes at least three to four hours for a single sample, most of this time being dedicated to pH stabilization process.

**Fig. 1:**
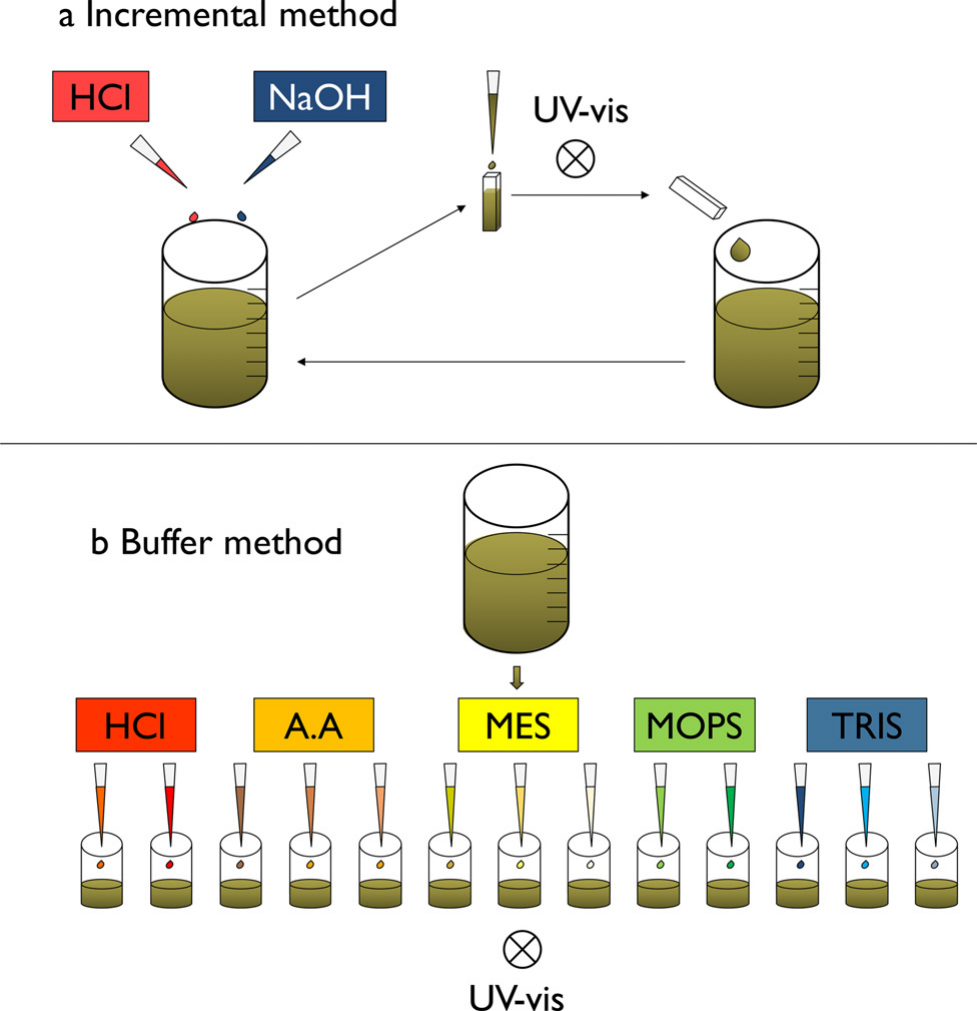
Diagram of the experimental setup for the “incremental method” (a) and the “buffer method” (b).

**Titration protocol using pH buffers.** The principle of the newly developed “buffer method” is to split the DOM solution into several aliquots (13 in the present study) and to adjust their pH using pH buffers. The pH of two aliquots was adjusted by HCl addition at pH 3 and 3.4. The pH of the 11 other aliquots was fixed using the buffer solutions, details of these buffers are listed in Table 1. Each aliquots contained a buffer solution at a concentration of 10^−3^ mol L^−1^. Absorbance was measured once the pH of each aliquot stabilized. This protocol was illustrated in Fig. 1b. Considering 10 mins for pH adjustment per sample (with HCl and buffer), 15 extra mins for pH stabilization, and manual absorbance measurements, this experimental setup only takes at most one to one and a half-hour. The UV-vis spectra of 10^−3^ mol L^−1^ buffer solutions in 10 mM NaCl in the absence of DOM were also recorded.

## Application example

**Fig. 2:**
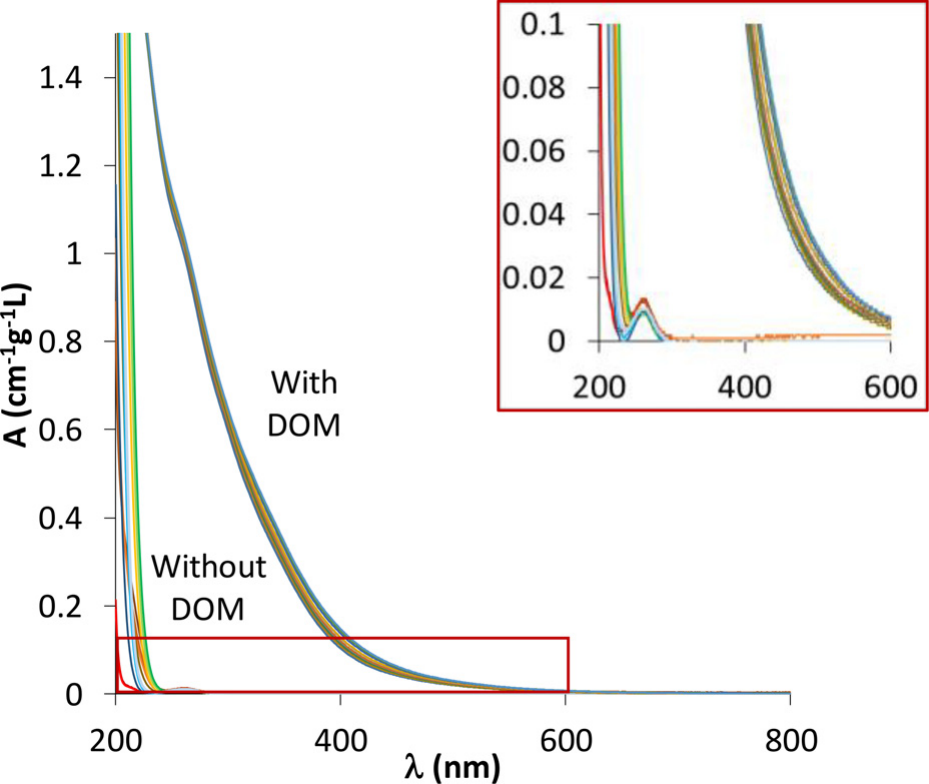
Absorbance spectra of buffer solutions (1 mM) in 10 mM NaCl, in the absence (colored lines) or the presence of 25mgC L^−1^ DOM (black lines).

**Absorbance of the buffer solutions.** The optical property of each buffer was investigated in a blank buffer solution in 10 mM NaCl, i.e. in the same conditions as the titrated DOM solution. Results are shown in Fig. 2. For wavelengths lower than 240 nm, a high absorbance was recorded for all of the buffer solutions, which is also observed for the background electrolyte solution (NaCl only). For some buffers, a slight absorption peak is observed within the 240 and 290 nm range with a maximum absorbance of 0.014 cm^−1^g^−1^L^−1^, which is very small and represents less than 1% (0.7% on average) of the absorbance of the corresponding samples containing 25 mgC L^−1^ DOM (Fig. 2). As the main wavelengths of interest, for DOM characterization, are centered around 270 and 370 [3], it is safe to consider that the buffer molecules do not contribute significantly to the absorbance of the DOM-containing samples.

**Fig. 3:**
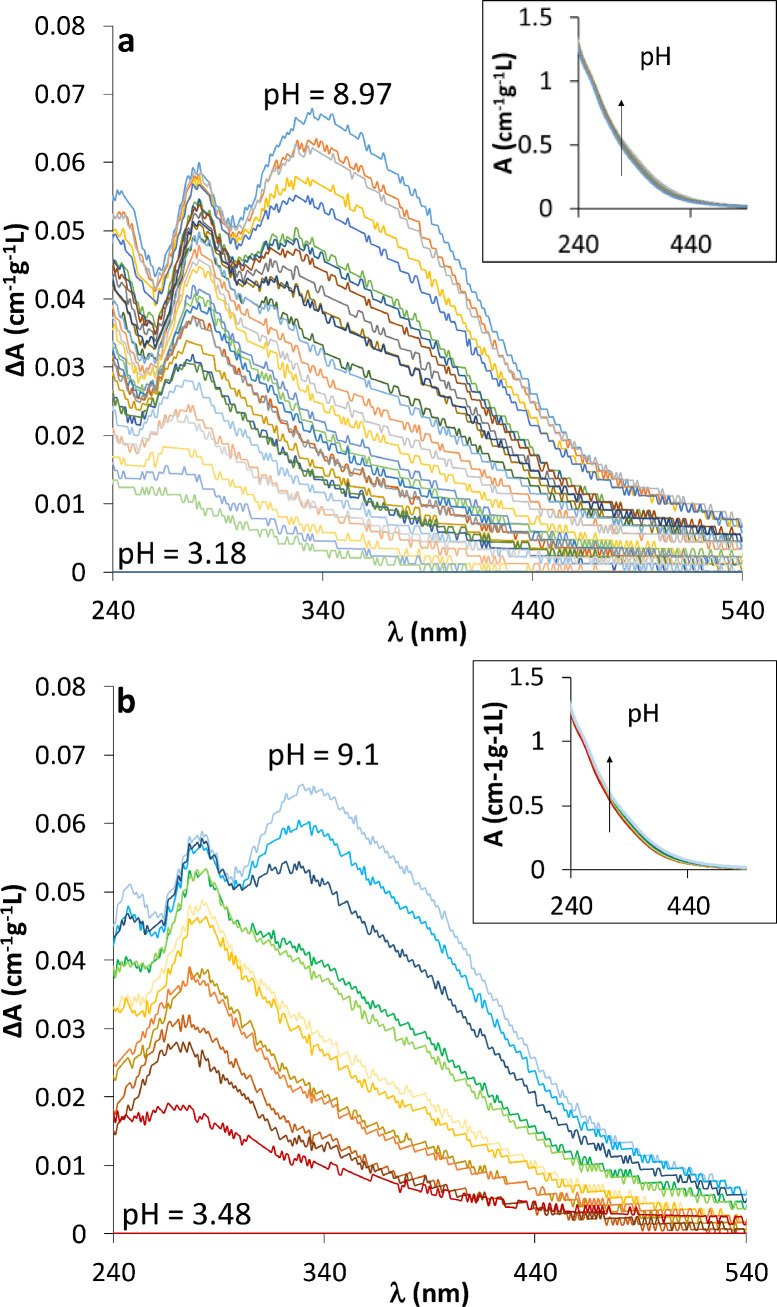
Relative absorbance (∆A) measurement with the incremental (a) and buffer (b) methods. Inserts in each figure are the corresponding raw absorbance (A).

**Spectrophotometric titration results.** The absorbance of DOM samples slightly varies with pH (Fig. 2) [3]. For visual ease, the absorbance spectra (A) were presented as the variation of absorbances (∆A) with respect to a reference spectrum as a function of the wavelength (λ) for both incremental and buffer methods in Fig. 3a and 3b, respectively. Their corresponding raw absorbances are presented in inserts in each figure. The sample at pH = 3 was taken as a reference to determine ∆A values:

(1) $$\Delta A_{\lambda }=A_{\lambda ,i}-\;A_{\lambda ,pH=3}$$

The absorbance measurement, with both incremental and buffer methods, presents a typical DOM absorbance spectra; the absorbance monotonously and progressively decreases with wavelength and increases with pH [3]. With both methods (Fig. 3 a and b) two peaks appear. The first one, centered around 270, is major at all pH values, wheras the second one, centered around 370, is smaller and appears as a shoulder of the first one, only for circumneutral to alkaline solutions (i.e for pH > 7). Comparing the two sets of ∆A, none difference stands out suggesting that the selected buffers do not affect the optical acid-base properties of the tested sample within the considered wavelength range.

**Fig. 4:**
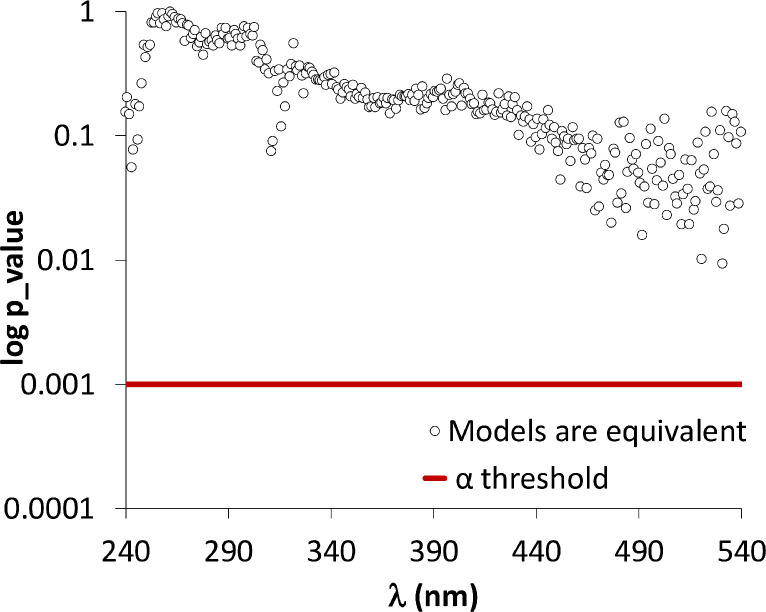
Evolution of p-values versus the wavelength in the regression analysis between the incremental and buffer methods. P-values correspond to a slope comparison using a Student's t-test. The significance threshold α is 0.001.

**Validation.** To verify that the use of buffer does not significantly impact the measured wavelength, a statistical validation was performed. The datasets of the incremental and the buffer methods were compared, at each wavelength, through mean and regression comparisons. The means of the incremental and the buffer ∆A were statistically compared by Student's t-test (p<.001) for each wavelength. After hypothesis verification, none of the wavelengths present a significant difference between the data drawn from the incremental and the buffer methods. Since comparing the means for each wavelength set could omit other information such as the variation, two linear models were developed to explain the correlation between absorbance and pH: one for the “incremental method” and other for the “buffer method”. The slope of the two models were then compared by a Student's t-test. None significant difference was underlined for all of the considered wavelengths (p>.001) when comparing the two models representing the sample pH stabilization method. This result suggests that the sample preparation has no significant impact on the absorbance variation (Fig. 4).

**Final remark.** This study brings forth a new experimental setup to characterize the acid-base properties of DOM by spectrophotometry acid base titration. Using the “buffer method”, an automatable, faster and reliable protocol, characterizing DOM at environmentally relevant concentrations becomes less tedious. Considering a coupling between a spectrophotometer and an automatic sampler, this method would allow an automation of the acid-base titration by UV-vis spectrophotometry, thus reducing the laboratory work period to a minimum. The disadvantages of this new method are only minor as it requires (i) additional chemicals (i.e. pH-buffers) associated with limited cost and (ii) preparation of pH-buffer stock solutions, which could be used for other numerous analyses. Multiplying the characterization of unknown DOM will help create and feed a data bank that will allow a better understanding of the acid-base properties of DOM and, indirectly, of its reactivity towards metal contaminants in the environment.

## Declaration of Competing Interest

The authors of this article declare that they have no conflict of interests.
